# Tissue or liquid rebiopsy? A prospective study for simultaneous tissue and liquid NGS after first‐line EGFR inhibitor resistance in lung cancer

**DOI:** 10.1002/cam4.6870

**Published:** 2023-12-22

**Authors:** Yen‐Ting Lin, Chao‐Chi Ho, Wei‐Hsun Hsu, Wei‐Yu Liao, Ching‐Yao Yang, Chong‐Jen Yu, Tzu‐Hsiu Tsai, James Chih‐Hsin Yang, Shang‐Gin Wu, Chia‐Lin Hsu, Min‐Shu Hsieh, Yen‐Lin Huang, Chia‐Ling Wu, Jin‐Yuan Shih

**Affiliations:** ^1^ Graduate Institute of Clinical Medicine National Taiwan University College of Medicine Taipei Taiwan; ^2^ Department of Medicine National Taiwan University Cancer Center Taipei Taiwan; ^3^ Department of Internal Medicine National Taiwan University Hospital and National Taiwan University College of Medicine Taipei Taiwan; ^4^ Department of Medical Research National Taiwan University Hospital Taipei Taiwan; ^5^ Department of Internal Medicine National Taiwan University Hospital Hsin‐Chu Branch Hsin‐Chu Taiwan; ^6^ Department of Medical Oncology National Taiwan University Cancer Center Taipei Taiwan; ^7^ Department of Oncology National Taiwan University Hospital Taipei Taiwan; ^8^ Graduate Institute of Oncology National Taiwan University College of Medicine Taipei Taiwan; ^9^ Department of Pathology National Taiwan University Hospital Taipei Taiwan; ^10^ Department of Pathology National Taiwan University Cancer Center Taipei Taiwan; ^11^ ACT Genomics Taipei Taiwan

**Keywords:** EGFR mutation, EGFR‐TKI resistance, next‐generation sequencing, NSCLC

## Abstract

**Introduction:**

According to current International Association for the Study of Lung Cancer guideline, physicians may first use plasma cell‐free DNA (cfDNA) methods to identify epidermal growth factor receptor (EGFR) tyrosine kinase inhibitor (TKI)‐resistant mechanisms (liquid rebiopsy) for lung cancer. Tissue rebiopsy is recommended if the plasma result is negative. However, this approach has not been evaluated prospectively using next‐generation sequencing (NGS).

**Methods:**

We prospectively enrolled patients with lung cancer with first‐line EGFR‐TKI resistance who underwent tissue rebiopsy. The rebiopsied tissues and cfDNA were sequenced using targeted NGS, ACTDrug®+, and ACTMonitor®Lung simultaneously. The clinicopathological characteristics and treatment outcomes were analyzed.

**Results:**

Totally, 86 patients were enrolled. Twenty‐six (30%) underwent tissue biopsy but the specimens were inadequate for NGS. Among the 60 patients with paired tissue and liquid rebiopsies, two‐thirds (40/60) may still be targetable. T790M mutations were found in 29, including 14 (48%) only from tissue and 5 (17%) only from cfDNA. Twenty‐four of them were treated with osimertinib, and progression‐free survival was longer in patients without detectable T790M in cfDNA than in patients with detectable T790M in cfDNA (*p* = 0.02). For the 31 T790M‐negative patients, there were six with mesenchymal–epithelial transition factor (MET) amplifications, four with ERBB2 amplifications, and one with CCDC6‐RET fusion. One with MET amplification and one with ERBB2 amplification responded to subsequent MET and ERBB2 targeting agents respectively.

**Conclusions:**

NGS after EGFR‐TKI resistance may detect targetable drivers besides T790M. To do either liquid or tissue NGS only could miss patients with T790M. To do tissue and liquid NGS in parallel after EGFR‐TKI resistance may find more patients with targetable cancers.

## 1 INTRODUCTION

Epidermal growth factor receptor (EGFR) mutations are the most common driver mutation in lung cancer.^1^ First‐generation EGFR‐tyrosine kinase inhibitors (TKIs) gefitinib and erlotinib, second‐generation EGFR‐TKI afatinib and dacomitinib, and third‐generation EGFR‐TKI osimertinib are approved as first‐line treatment for advanced non‐small cell lung cancer (NSCLC) with activating EGFR mutations. The median overall survival (OS) of patients with advanced lung cancer treated with first‐line gefitinib, erlotinib, or afatinib is more than 3 years.^2^ However, most patients still experience disease progression after EGFR‐TKI therapy. Acquired EGFR T790M mutation, which accounts for around 50% of mutations, is the most common mechanism of resistance for first‐ and second‐generation EGFR‐TKIs.^3^ Third‐generation EGFR‐TKI osimertinib overcomes T790M mutation and is the drug of choice for patients with T790M‐positive disease.^4^ Osimertinib resistance is more heterogeneous; on target‐acquired resistance, EGFR C797S and EGFR amplification only contribute to a minority of resistance.^5^, ^6^ Bypass pathway activation, such as mesenchymal–epithelial transition factor (MET) amplification,^7^ Erb‐b2 receptor tyrosine kinase 2 (ERBB2) amplification,^8^ V‐Raf murine sarcoma viral oncogene homolog B (BRAF) mutations,^9^ Kirsten rat sarcoma virus (KRAS) mutations,^10^ and oncogenic fusions^11^, ^12^ are also important mechanisms of resistance.^13^, ^14^ In addition to EGFR inhibition, there is clinical evidence that inhibiting MET amplification,^15^, ^16^, ^17^ ERBB2 amplification,^18^ BRAF mutation,^19^ rearranged during transfection (RET) fusion,^11^, ^20^ and anaplastic lymphoma kinase (ALK) fusion^12^ may be effective in controlling EGFR‐TKI‐resistant lung cancers. Next‐generation sequencing (NGS) is a powerful tool to detect dozens or hundreds of possible oncogenic drivers at a time.^21^ Originally, NGS only applied to cancer tissue; however, liquid NGS (analysis of circulating cell‐free DNA [cfDNA] or circulating tumor DNA by NGS) is now available commercially. Liquid NGS is less invasive, and the turnaround time is less than that of tissue biopsy.^22^ According to current International Association for the Study of Lung Cancer (IASLC) guideline,^23^ physicians may use cfDNA plasma testing in patients with lung adenocarcinoma with progression to EGFR‐TKIs. Tissue rebiopsy is recommended if the plasma result is negative. The recommendation is based on trials and cohorts using polymerase chain reaction‐based detection systems for EGFR.^24^, ^25^, ^26^, ^27^ However, this approach has not been validated in a prospective cohort in the era of tissue and liquid NGS. Thus, we conducted a prospective study to evaluate the efficacy of both tissue NGS (tissue rebiopsy) and liquid NGS (liquid rebiopsy) at the time of cancer progression after first‐line EGFR‐TKI treatment in patients with advanced NSCLC.

## 2 MATERIALS AND METHODS

### 2.1 Patients

This prospective study was conducted at a tertiary referral center, National Taiwan University Hospital (NTUH), Taipei, Taiwan. Patients with advanced NSCLC with activating EGFR mutations treated with first‐line EGFR‐TKI were eligible after disease progression if their cancers could be rebiopsied. This study was approved by the NTUH Research Ethics Committee (201905056RIFD) and all patients provided informed consent prior to enrollment.

### 2.2 Prior EGFR‐TKI and sequential treatment assessment

Patient characteristics, including age, sex, smoking history, cancer cell type, comorbidities, and cancer stage,^28^ as well as treatment details regarding first‐line EGFR‐TKI, sequential anticancer treatment, tumor response, progression‐free survival (PFS), survival status, and OS, were recorded. Experienced study nurses employed questionnaire on the smoking status and comorbidities. The treatment dose and schedules were per clinicians' choice. PFS was defined as the period between the start of a treatment (such as EGFR‐TKIs, or sequential treatments) to disease progression or death or date of last follow‐up visit. OS was defined as the period between study enrollment to death or last follow‐up. Patients were followed up by imaging studies (computed tomography scan and/or magnetic resonance imaging) every 3 months and as necessary. Tumor response was evaluated using the Response Evaluation Criteria in Solid Tumors criteria 1.1.^29^ As the mechanisms of resistance and sequential treatments are different between first‐ or second‐generation EGFR‐TKI and osimertinib, patients receiving first‐line osimertinib were analyzed separately. Targetable acquired resistance mechanisms were defined as the presence of EGFR T790M,^4^ BRAF V600E,^19^ BRAF fusion,^30^ RET fusion,^11^ ALK fusion,^12^ c‐ros oncogene 1 (ROS1) fusion,^31^ MET exon 14 skipping,^32^ MET amplification,^15^, ^16^, ^17^ or ERBB2 amplification.^18^

### 2.3 NGS after EGFR‐TKI resistance

All enrolled patients underwent tissue biopsy NGS and liquid biopsy NGS after EGFR‐TKI resistance. We used ACTDrug®+ (ACTGenomics, Taipei, Taiwan) for tissue biopsy, and ACTMonitor®Lung (ACTGenomics, Taipei, Taiwan) for liquid biopsy. ACTDrug®+ includes a DNA NGS that sequences 40 potentially actionable genes and an RNA NGS, ACTFusion™, capable of detecting 13 fusion genes. ACTMonitor®Lung sequences hotspots 11 lung cancer‐related genes from patients' cfDNA. The details of the NGS study methods and decoded genes are provided in Data S1 and Table S1.

### 2.4 Statistical analysis

Continuous variables are reported as medians with interquartile ranges (IQR). Categorical data was compared using the chi‐squared test and survival analyses were performed using the Kaplan–Meier method and the log‐rank test. Statistical significance was set at *p* value of less than 0.05. All statistical analyses were performed using the Statistical Package for Social Sciences, version 18.0 (SPSS, Inc., Chicago, IL, USA).

## 3 RESULTS

### 3.1 Patient demographic and clinical characteristics

**FIGURE 1:**
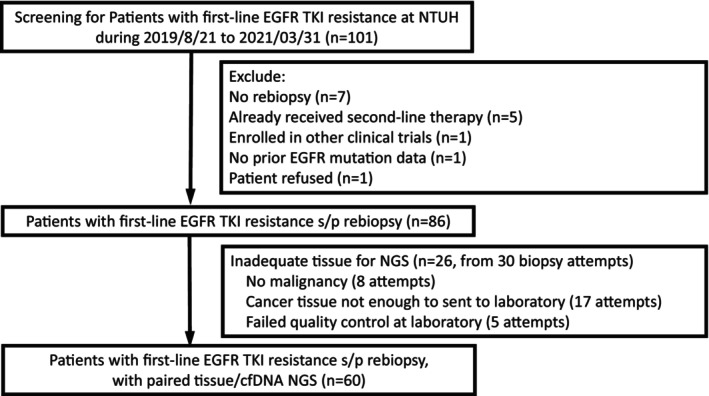
Study flow chart.

Eighty‐six patients who received tissue and liquid rebiopsy were enrolled (Figure 1). Patient demographic data are presented in Table 1. All of them were Asian. The details of rebiopsy are listed in Table S2. Twenty‐six (30%) of them underwent tissue biopsy but their specimens were inadequate for NGS. Four of these patients had a failed tissue biopsy for NGS twice. Patients with inadequate tissue specimens for NGS tended to be older (median age, 67.7 vs. 62.5 year‐old, *p* = 0.04, Table 1). Among the 93 rebiopsy attempts, 26 patients with inadequate tissue for NGS underwent 30 biopsy attempts and 13 (43%) underwent CT‐guided biopsy, while 60 with adequate tissue for NGS underwent 63 biopsy attempts and 11 (18%) underwent CT‐guided biopsy (*p* = 0.008). Among the 60 patients with paired tissue and liquid NGS, 14 received gefitinib, 28 received erlotinib, 16 received afatinib, and 2 received osimertinib as first‐line EGFR‐TKI (Table 1). The response rate (RR) to first‐line EGFR‐TKI in the cohort was 68% and the disease control rate (DCR) was 95%. The median first‐line EGFR‐TKI PFS was 11.7 (95% confidence interval [CI], 8.2–15.1) months.

**TABLE 1:** Demographic data (*n* = 86).

Variable	Total patients (*n* = 86)	Patients with paired NGS results (*n* = 60)	Patients with liquid NGS result only (*n* = 26)	*p*
Median age at advanced disease (year‐old) (IQR)	64.1 (57.8–72.1)	62.5 (55.9–70.3)	67.7 (62.0–74.2)	0.04
Male	32 (37%)	22 (37%)	10 (39%)	0.87
Patients who never smoked	71 (83%)	49 (82%)	22 (85%)	0.74
Adenocarcinoma	77 (90%)	52 (87%)	25 (96%)	0.19
Primary EGFR mutation				0.28
Exon 19 deletion	37 (43%)	29 (48%)	8 (31%)	
L858R	45 (53%)	27 (45%)	18 (69%)	
G791X	1 (1%)	1 (2%)	0 (0%)	
L861Q	2 (2%)	2 (3%)	0 (0%)	
G724S+S768I	1 (1%)	1 (2%)	0 (0%)	
First‐line EGFR‐TKI				0.78
Gefitinib	18 (21%)	14 (23%)	4 (15%)	
Erlotinib	43 (50%)	28 (47%)	15 (58%)	
Afatinib	22 (26%)	16 (27%)	6 (23%)	
Osimertinib	3 (4%)	2 (3%)	1 (4%)	
Median follow‐up (month) (IQR)	18.3 (10.4–23.7)	18.6 (12.3–24.2)	17.2 (10.2–22.6)	0.72

Abbreviations: IQR, interquartile range; NGS, next‐generation sequencing; TKI, tyrosine kinase inhibitor.

### 3.2 Tissue biopsy NGS after EGFR‐TKI resistance

**FIGURE 2:**
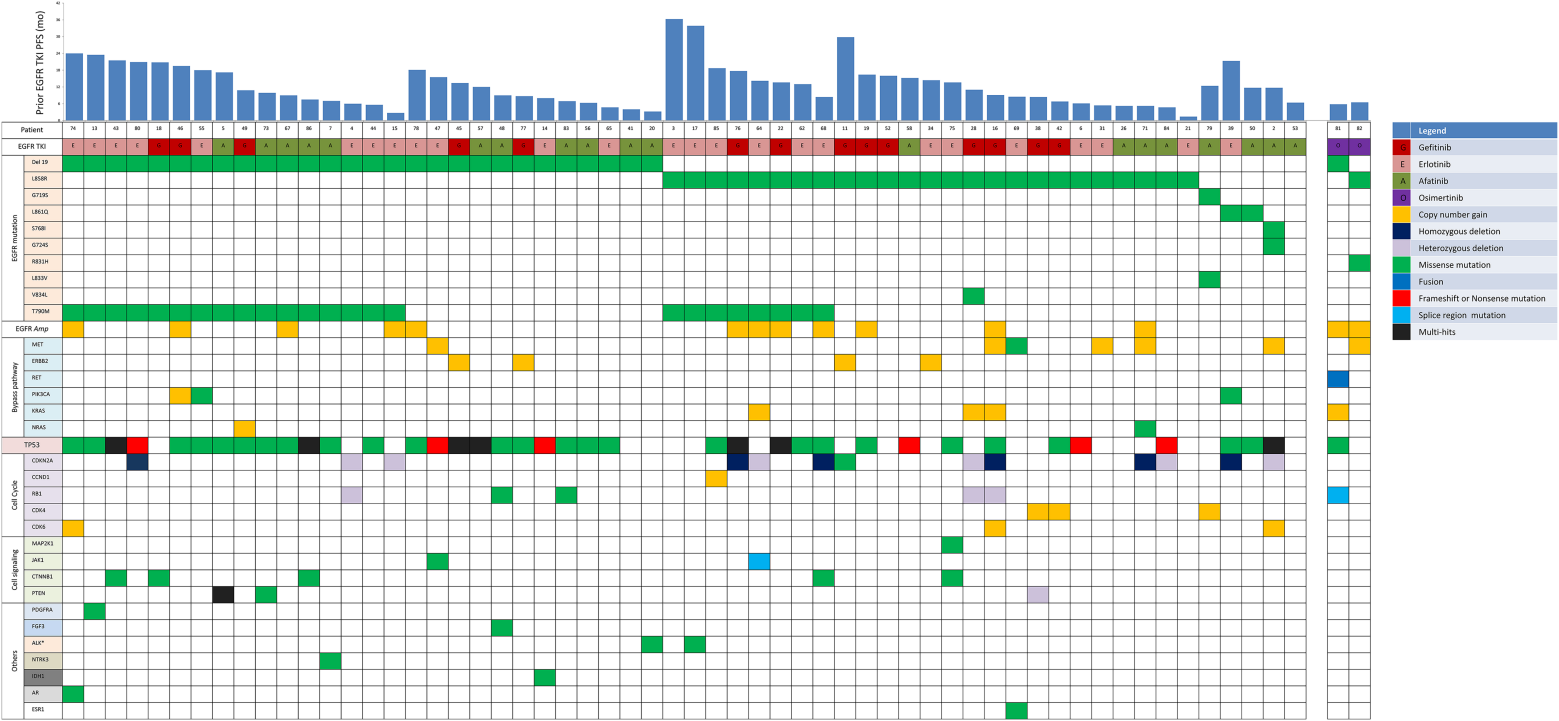
Tissue next‐generation sequencing result. Detected genetic alterations are grouped by the function. Bars above indicate prior EGFR‐TKI progression‐free survival (PFS). The two patients who received first‐line osimertinib are shown separately on the right. EGFR, epidermal growth factor receptor; TKI, tyrosine kinase inhibitor.

Tissue biopsy NGS results from 60 patients with adequate cancer tissue are shown in Figure 2. TP53 mutations were the most frequent co‐mutations (65%, 39 out of 60) besides EGFR. Of the 58 patients who received gefitinib, erlotinib, or afatinib as first‐line EGFR‐TKI, 24 (41%) T790M mutations were found using tissue NGS. Of the remaining 34 patients who were T790M‐negative, 4 had ERBB2 amplification, 3 had MET amplification, 3 had EGFR amplification, and 2 had both MET and EGFR amplification in addition to the original EGFR mutations. Of the 2 patients who received osimertinib as their first‐line EGFR‐TKI, 1 had CCDC6‐RET fusion and the other had both MET and EGFR amplification. Overall, 58% (35 out of 60) of patients had targetable drivers discovered by tissue NGS after first‐line EGFR‐TKI resistance.

### 3.3 Liquid biopsy NGS after EGFR‐TKI resistance

**FIGURE 3:**
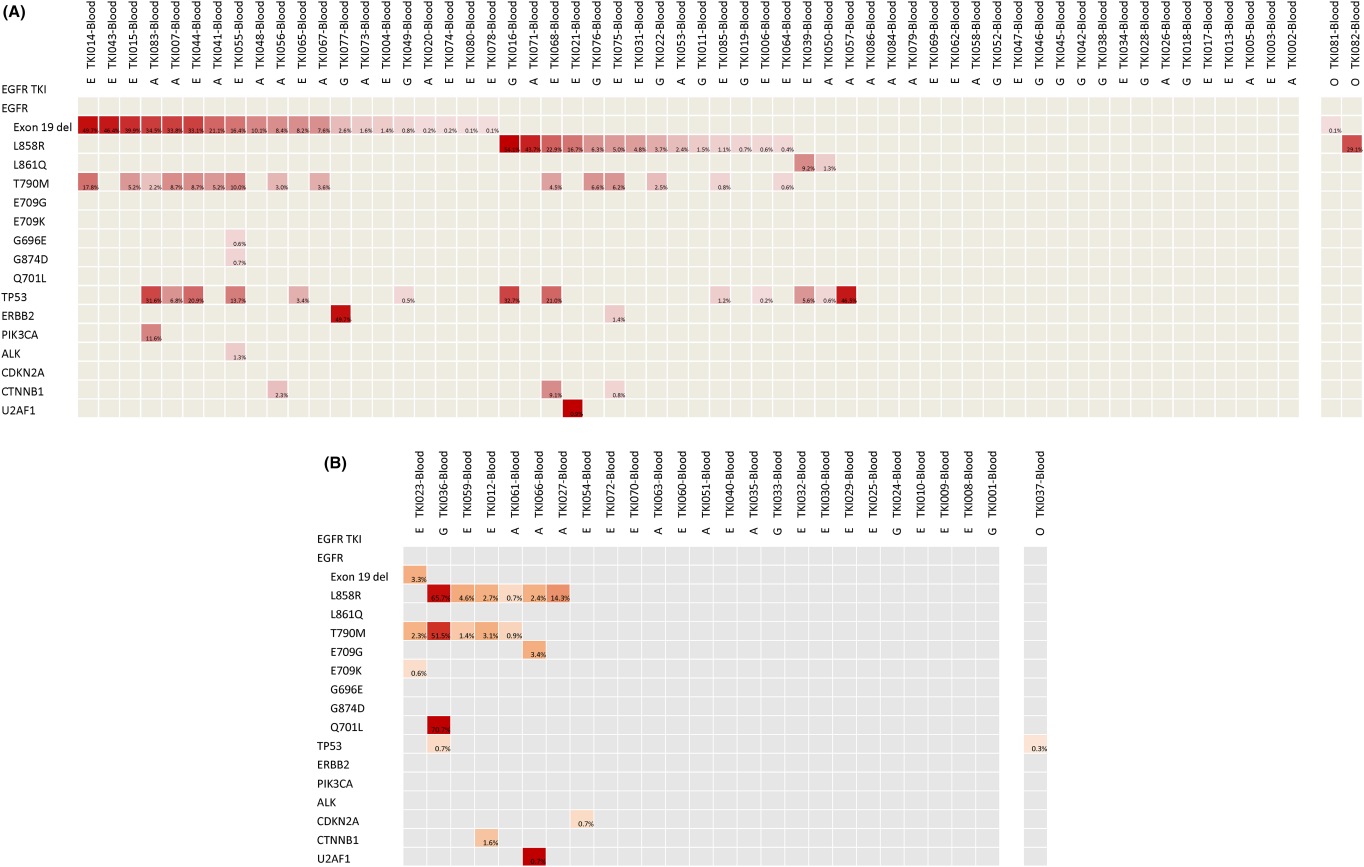
Liquid next‐generation sequencing (NGS) result. The depth of color represents the allele frequency detected. (A) Liquid NGS from the 60 patients with paired tissue and liquid NGS. The two patients who received first‐line osimertinb are shown separately on the right. (B) Liquid NGS from the 26 patients without paired tissue NGS. The one patient who received first‐line osimertinib is shown separately on the right. A, afatinib; E, erlotinib; EGFR, epidermal growth factor receptor; G, gefitinib; O, osimertinib; TKI, tyrosine kinase inhibitor.

The liquid biopsy NGS results from the 60 patients with paired tissue and liquid NGS are shown in Figure 3A. Of the 58 patients who received gefitinib, erlotinib, or afatinib, EGFR T790M was detected in 26% (15 out of 58) of the cfDNA samples. Five of them did not have the T790M mutation detected by tissue NGS. For the two patients who received first‐line osimertinib (TKI‐081 and TKI‐082), liquid rebiopsy NGS only revealed EGFR exon 19 deletion and L858R, respectively.

The liquid biopsy NGS results from the 26 patients without paired tissue and liquid NGS are shown in Figure 3B. The median OS of the 25 patients who received gefitinib, erlotinib, or afatinib was 20.4 (95% CI, 12.7–28.1) months. Among them, five T790M mutations were identified, and four patients received subsequent osimertinib with a median treatment duration of 8.4 (95% CI, 2.3–14.5) months. No other targetable driver was found by liquid NGS. For the patient who received first‐line osimertinib, only a TP53 mutation (N239_C242del) was found by liquid NGS after osimertinib resistance.

For all the 86 patients, 45 (52%) had EGFR mutations detected from the liquid NGS at first‐line EGFR‐TKI progression while the other 41 did not. The patient characteristics between EGFR mutations detected or not were listed in Table S3. Patients without EGFR mutation detected by liquid NGS at progression had a longer first‐line EGFR‐TKI PFS (median, 14.3 [95% CI, 12.1–16.6] vs. 9.1 [95% CI, 8.4–9.7] months, Figure S1A, *p* = 0.007). For patients with the EGFR mutation found in liquid NGS, 19 (42%) patients received subsequent osimertinib for T790M. For the patients without EGFR mutation found in liquid NGS (but with EGFR T790M found in their cancer tissues), 8 (17%) patients received subsequent osimertinib. Patients with EGFR mutation detected from liquid NGS had higher proportion to receive osimertinib for T790M (*p* = 0.02). The osimertinib PFS tended to be longer in the patients without EGFR mutation detected from liquid NGS (median, not reached vs. 12.1 [95% CI, 8.4–15.8] months, Figure S1B, *p* = 0.07). No significant OS difference was noted between patients with and without EGFR mutation detected from liquid NGS (median, 23.7 [95% CI, 15.1–32.3] vs. 30.1 [95% CI, 16.8–43.4] months, Figure S1C, *p* = 0.52).

### 3.4 Concordance between paired tissue and liquid biopsy NGS after EGFR‐TKI resistance

**FIGURE 4:**
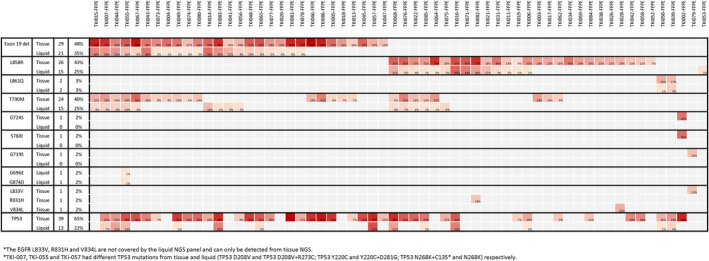
Concordance between tissue and liquid next‐generation sequencing.

The concordance between tissue and liquid NGS results is shown in Figure 4. The concordance rates between tissue and cfDNA were 87% for exon 19 deletions, 78% for L858R, 68% for T790M, and 57% for TP53, respectively.

### 3.5 Resistance profiles, subsequent treatments, and outcome in patients who received paired tissue and liquid biopsy NGS after EGFR‐TKI resistance

**FIGURE 5:**
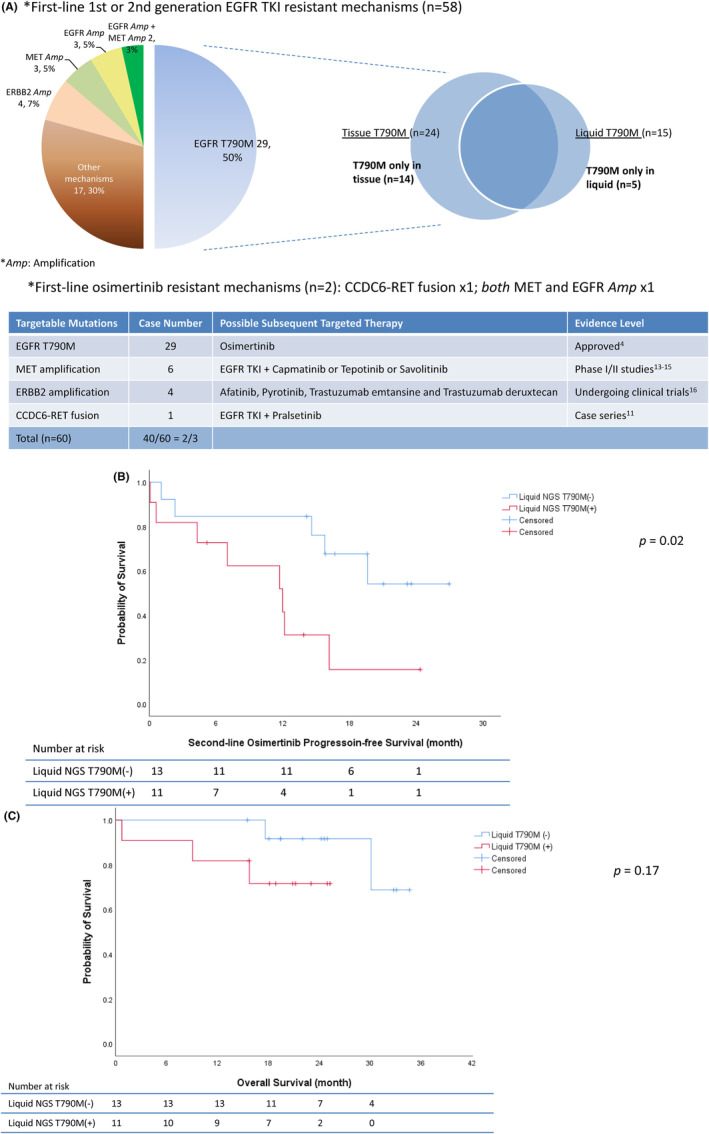
EGFR‐TKI resistant mechanisms and sequential treatment outcome. (A) Mechanisms of EGFR‐TKI resistance in this study. The possible subsequent targeted therapies were listed below. (B) Kaplan–Meier analyses of progression‐free survival for sequential osimertinib in patients with T790M‐positive mutations. The survivals between patients with positive and negative T790M mutations on liquid NGS are compared using the log‐rank test. (C) Kaplan–Meier analyses of overall survival for subsequent osimertinib treatment in patients with T790M‐positive mutations. The survivals between patients with positive and negative T790M mutations on liquid NGS are compared using the log‐rank test. *Amp*, amplification; EGFR, epidermal growth factor receptor; NGS, next‐generation sequencing; TKI, tyrosine kinase inhibitor.

Of the 58 patients who received first‐line gefitinib, erlotinib, or afatinib, T790M was detected in 29 (50%) patients. Five (17%) T790M mutations were detected only by liquid NGS, and 14 (48%) T790M mutations were detected only by tissue NGS (Figures 4 and 5A). Of the 29 patients who were T790M‐positive, 24 were subsequently treated with osimertinib. The PFS was longer in patients without detectable T790M in cfDNA (i.e., negative liquid NGS for T790M) (*n* = 13) than in patients with T790M detected in cfDNA (*n* = 11) (median, not reached vs. 12.0 months, *p* = 0.02) (Figure 5B). There may be a trend for longer OS after osimertinib treatment in patients without detectable T790M in cfDNA (median not reached, *p* = 0.17; Figure 5C). For the 29 patients who were T790M‐negative, four ERBB2 amplifications, three MET amplifications, and three EGFR amplifications were identified. An additional two patients were found to have both MET and EGFR amplifications (Figure 5A). For patients with MET amplification, one patient with EGFR L858R and MET amplification was treated with erlotinib in combination with capmatinib. The treatment response for erlotinib plus capmatinib was partial response (PR), and the PFS was 12.4 months. For patients with ERBB2 amplification, one patient received afatinib plus bevacizumab, with PR and a PFS of 7.5 months, after first‐line gefitinib progression. The other patients did not receive any MET inhibitors, amivantamab, or ERBB2 targeting agents. The presence of TP53 mutation or not, at EGFR‐TKI resistance did not affect patient's OS (median, 30.1 month vs. not reached, *p* = 0.60, Figure S2).

Patients who received first‐line osimertinib (*n* = 2) received sequential atezolizumab, bevacizumab, pemetrexed, and carboplatin. PFS was 5.3 and 4.6 months, respectively.

## 4 DISCUSSION

In this prospective study, we analyzed the cancer tissues and cfDNA of patients with lung cancer using NGS simultaneously after EGFR‐TKI resistance. Approximately 30% of patients did not have adequate cancer tissue for NGS, even after rebiopsy. By combining tissue and liquid NGS, two‐thirds of the patients (67%, 40 out of 60) may still have druggable targets after first‐line EGFR‐TKI resistance (38 out of 58 after a first‐ or second‐generation EGFR‐TKI and 2 out of 2 after osimertinib) (Figure 5A). EGFR T790M mutations were found in 50% of patients after first‐ or second‐generation EGFR‐TKI resistance. While tissue NGS revealed the majority of T790M mutations, liquid NGS detected an additional 17% of T790M mutations (Figure 5A). For patients subsequently treated with osimertinib, PFS was longer in patients with negative T790M by liquid NGS than in patients with T790M detected by liquid NGS (Figure 5B). Besides EGFR T790M, we found MET amplification, ERBB2 amplification and RET fusion as possible targetable drivers. One with MET amplification and one with ERBB2 amplification responded to subsequent MET and ERBB2 targeting agents respectively.

Tissue rebiopsy with NGS study is ideally performed at the time of EGFR‐TKI resistance. However, tissue rebiopsy may not always be feasible during disease progression. In this prospective study, we found CT‐guided biopsy had less chance to obtain enough tissue for NGS study. It may be related to the small amount of specimens taken by CT‐guided biopsy. Approximately 30% of the patients (*n* = 26) still did not have adequate tissue for NGS after tissue rebiopsy procedures, including four patients with multiple attempts for rebiopsy (Table S2). However, we found five patients with EGFR T790M mutations using liquid NGS among them. Four of them received subsequent osimertinib and the median treatment duration was 8.4 months. Although treatment duration was numerically shorter than the PFS in the paired NGS patients with detectable T790M in blood, those patients were elder, and 8.4 months may still be clinically significant. These findings highlight the importance of performing a liquid rebiopsy. In prior studies, the rate of failure to perform tissue NGS ranged from 7% to as much as 50%, depending on the study design, biopsy method, location, and genetic analysis platform.^33^, ^34^, ^35^, ^36^ In the real world, patients experiencing treatment failure may be symptomatic and weak, and the tumors may not always be easily approachable.^37^ Moreover, tumor heterogeneity is not uncommon in advanced cancers, especially after drug resistance.^38^ Liquid NGS could reflect the complete genetic status of a tumor compared with a single tissue biopsy.^22^

On the other hand, liquid rebiopsy without tissue rebiopsy after EGFR‐TKI resistance missed approximately half of EGFR T790M mutations in this study (Figure 5A). Moreover, the PFS for sequential osimertinib treatment was longer for patients with negative T790M mutation liquid NGS for than for patients with detectable plasma T790M mutations. This may be because a “non‐shedding” tumor may be less extensive, and its behavior may be less aggressive, than that of a shedding tumor. Tissue genetic analysis is necessary to identify plasma T790M‐negative non‐shedding tumors. Our finding was consistent with earlier studies with mixed treatment‐naive and treatment‐resistant lung cancer.^35^, ^36^ In this prospective study, we can see the advantages of taking both tissue and liquid rebiopsy. Combined tissue and liquid rebiopsy found more patients with EGFR T790M mutation than only tissue or liquid rebiopsy (Figure 5A), leading to more subsequent osimertinib treatment. Paired tissue and liquid NGS may influence subsequent treatment and patient outcome. Our findings support to use tissue and liquid rebiopsy in parallel after EGFR‐TKI resistance in lung cancer.

Besides EGFR T790M, MET amplifications and ERBB2 amplifications are well‐documented acquired resistance after EGFR‐TKI.^3^, ^5^ Combining EGFR inhibition and MET inhibition showed anticancer activity in EGFR‐TKI‐resistant MET‐amplified lung cancers in clinical trials.^15^, ^16^ Clinical trials with afatinib, pyrotinib, trastuzumab emtansine, and trastuzumab deruxtecan are being evaluated for their efficacy against EGFR‐TKI‐resistant ERBB2 amplified lung cancers.^39^ To evaluate acquired resistance to EGFR‐TKI, a single test for EGFR mutation is not sufficient and NGS is the preferred method for genetic testing after EGFR‐TKI resistance. Besides tissue and liquid NGS from blood, there are increasing evidence that liquid NGS from pleural effusion,^40^, ^41^, ^42^ ascites,^43^ and cerebrospinal fluid^44^ can provide information for tumor genetics and subsequent treatments. Body fluid NGS is particularly of value, especially in patients with only peritoneal or pleural metastases or leptomeningeal carcinomatosis.

In this study, the concordance rate of TP53 between tissue and liquid NGS was only 57% and TP53 was discovered more from tissue NGS (Figure 4). The OS after EGFR‐TKI resistance was not associated with the TP53 status at rebiopsy. The concordance rate of nondriver mutations between tissue and liquid NGS in after EGFR‐TKI resistance lung cancer is seldom reported. The panel of tissue and liquid of NGS may be different, and the methodology of liquid NGS may limit its ability to detect genetic alterations. In our study, the tissue NGS panel ACTDrug®+ used cancer tissue RNA to detect oncogenic fusion, while the liquid NGS panel ACTMonitor®Lung only tests blood cfDNA, and fusion may not be detected. RNA‐based NGS is preferred over DNA‐based NGS for oncogenic fusion detection.^45^ Although a plasma cell‐free RNA assay has been established for lung cancer,^46^ it has not been widely used. This predicament occurs in current popular NGS platforms, such as FoundationOneLiquid (Foundation Medicine, Cambridge, MA, USA) and Guardant360 (Guardant Health, Redwood City, CA, USA).

The use of NGS for driver mutation screening in lung cancer has not been routinely recommended. Current ESMO guideline suggested to use tumor or plasma NGS only to detect the ESMO Scale for Clinical Actionability of molecular Targets (ESCAT) level I mutations (i.e., mutations with approved targeted therapy).^21^ In the Asia‐Pacific region, NGS for metastatic cancers are reimbursed in Japan, Australia, and South Korea, but not in Thailand, Hong Kong, Singapore, Malaysia, and the Philippines. The Asia‐Pacific Oncology Drug Development Consortium (APODDC) recommended small‐panel, multiplex‐gene NGS with adequate coverage of key alterations for NSCLC in 2023.^47^ In Taiwan, NGS study for lung cancer is not reimbursed as well. Actually, the Taiwan National Health Insurance (NHI) only reimbursed second‐line osimertinib from EGFR T790M detected by tissue methods (plasma methods *not* allowed). In the real‐world practice, we often use hot spot analysis, such as Roche Cobas V2, rather than an NGS to detect EGFR mutations at both lung cancer diagnosis, and resistance to an EGFR‐TKI. We seldom do both liquid and tissue NGS simultaneously, because of the cost, as well as lack of evidence of benefit. Thus, we conducted this prospective study to evaluate how much we could gain by doing tissue and liquid NGS in parallel after EGFR‐TKI resistance. If we assume all EGFR T790M and RET fusion discovered from NGS in this study can be detected by other cheaper PCR‐based analyses, 30 (50%) patients' cancer will be targetable (Figure 5A). Taking current reimbursement criteria in Taiwan (plasma T790M result *not* accepted and pralsetinib *not* reimbursed) into account, only 24 (40%) patients could receive subsequent targeted therapy covered by our NHI. Considering the cost of both NGS and the sequential treatment, whether all patients should receive NGS is still debated. In the upfront testing, NGS testing was reported to be associated with substantial cost savings and shorter time‐to‐test results in the United States,^48^ but whether the result can be generalized to the countries with limited health resources, and to evaluate EGFR‐TKI resistance, is still uncertain. In the real world, an EGFR test should be ordered after EGFR‐TKI resistance, in order to detect T790M mutation for sequential osimertinib treatment.^23^ Whether the EGFR test should be a more economic PCR‐based hotspot analysis, or a comprehensive NGS in order to find more possible targets, depends on the resources available. The possibility of tissue rebiopsy, the cost of NGS and subsequent drugs, and the availability of clinical trials should be taken into consideration. Hopefully in the future, as the advance of NGS technology (with decreasing cost in the future) and drug development (with more Level I targets and affordable drugs), we can do tissue and liquid NGS in parallel after EGFR‐TKI resistance, to give patients opportunities for *more* precision medicine.

This study had several limitations. First, this was a single‐center prospective study and the number of patients was relatively limited. Second, osimertinib has been approved as the first‐line treatment for advanced EGFR‐mutant lung cancer, but the study was conducted prior to its reimbursement by the National Health Insurance in Taiwan; thus, we only had three first‐line osimertinib‐treated patients (two with paired tissue and liquid NGS and one with liquid NGS only). However, we discovered two druggable first‐line osimertinib‐resistant mechanisms: RET fusion and MET amplification. Third, not all genetic alterations were discovered by our NGS panels. However, the NGS panels have been able to identify most potentially targetable drivers. Fourth, we did not sent the cancer tissues before EGFR‐TKI treatment for NGS. We cannot confirm all the resistant mechanisms discovered were acquired. However, the cohort responded well to prior EGFR‐TKIs (RR 68%, DCR 95%, PFS 11.7 months).

In conclusion, NGS after EGFR‐TKI resistance may provide a greater chance of detecting potential druggable targets than solely testing for EGFR T790M. Performing liquid NGS at EGFR‐TKI progression only, without tissue genetic analysis, may cause an underestimation of a large number of patients with T790M mutations who may benefit from second‐line osimertinib treatment, and vice versa. Doing tissue and liquid NGS in parallel after EGFR‐TKI resistance may find more patients with targetable cancers.

### 4.1 Statements and declarations

The results of this study have been presented, in part, at the at the 2021 Annual Congress of Taiwan Society of Pulmonary and Critical Care Medicine in Taichung, Taiwan, December 11, 2021 and European Lung Cancer Congress on April 03, 2022 (*Ann Oncol*. 2022;33(suppl_2):S58).

## AUTHOR CONTRIBUTIONS

**Yen‐Ting Lin:** Conceptualization (equal); data curation (equal); formal analysis (lead); investigation (equal); methodology (equal); resources (equal); visualization (equal); writing – original draft (lead). **Chao‐Chi Ho:** Conceptualization (equal); investigation (equal); methodology (equal); resources (equal); writing – review and editing (equal). **Wei‐Hsun Hsu:** Conceptualization (equal); investigation (equal); methodology (equal); resources (equal); writing – review and editing (equal). **Wei‐Yu Liao:** Conceptualization (equal); investigation (equal); methodology (equal); resources (equal); writing – review and editing (equal). **Ching‐Yao Yang:** Conceptualization (equal); investigation (equal); methodology (equal); resources (equal); writing – review and editing (equal). **Chong‐Jen Yu:** Conceptualization (equal); investigation (equal); methodology (equal); resources (equal); writing – review and editing (equal). **Tzu‐Hsiu Tsai:** Conceptualization (equal); investigation (equal); methodology (equal); resources (equal); writing – review and editing (equal). **James Chih‐Hsin Yang:** Conceptualization (equal); investigation (equal); methodology (equal); resources (equal); writing – review and editing (equal). **Shang‐Gin Wu:** Conceptualization (equal); investigation (equal); methodology (equal); resources (equal); writing – review and editing (equal). **Chia‐Lin Hsu:** Conceptualization (equal); investigation (equal); methodology (equal); resources (equal); writing – review and editing (equal). **Min‐Shu Hsieh:** Investigation (equal); resources (equal); writing – review and editing (equal). **Yen‐Lin Huang:** Investigation (equal); resources (equal); writing – review and editing (equal). **Chia‐Ling Wu:** Data curation (equal); formal analysis (equal); investigation (equal); methodology (equal); resources (equal); software (equal); visualization (equal); writing – review and editing (equal). **Jin‐Yuan Shih:** Conceptualization (lead); data curation (equal); formal analysis (equal); funding acquisition (lead); investigation (equal); methodology (equal); project administration (lead); resources (equal); supervision (lead); writing – review and editing (lead).

## FUNDING INFORMATION

This study was supported by ACT Genomics. ACT Genomics only provided NGS tests and analyses. Investigators were responsible for study conception, design, protocol writing, data collection, interpretation, and manuscript preparation.

## CONFLICT OF INTEREST STATEMENT

YTL has received speaking honoraria from ACT Genomics, Amgen, AstraZeneca, Boehringer Ingelheim, Bristol‐Myers Squibb, Chugai Pharmaceutical, Eli Lilly, Illumina, Janssen, Manudipharma, Merck, Merck Sharp & Dohme, Novartis, Pfizer, Roche, and Takeda. WHH has received speaking honoraria from ACT Genomics, AstraZeneca, Chugai Pharmaceutical, Eli Lilly, Janssen, Merck, Ono Pharmaceutical, Pfizer, Roche, and Takeda. WYL has received speaking honoraria from AstraZeneca, Bayer, Boehringer Ingelheim, Bristol‐Myers Squibb, Chugai Pharmaceutical, Eli Lilly, Johnson & Johnson, MSD Oncology, Novartis, Pfizer, and Roche. JCHY has received research grant from AstraZeneca; institutional fee for advisory or consultancy services from Amgen, AstraZeneca, Bayer, Boehringer Ingelheim, Bristol‐Myers Squibb, Daiichi Sankyo, Eli Lilly, Merck, Merck Sharp & Dohme, Novartis, Roche/Genentech, Takeda Oncology, Yuhan Parmaceuticals, and JNJ; advisory or consultancy services from Ono Pharmaceuticals and Pfizer; and institutional fee for advisory services from advisory services from Puma Technology, Gilead, and GSK. SGW has received speaking honoraria from Amgen, AstraZeneca, Boehringer Ingelheim, Chugai Pharmaceutical, Eli Lilly, Janssen, Novartis, Pfizer, Roche, and Takeda. JYS has received research grant from Roche; speaking honoraria from ACT Genomics, Amgen, AstraZeneca, Bayer, Boehringer Ingelheim, Bristol‐Myers Squibb, Chugai Pharmaceutical, CStone Pharmaceuticals, Eli Lilly, Janssen, Genconn Biotech, Manudipharma, Merck Sharp & Dohme, Novartis, Ono Pharmaceutical, Orient EuroPharma, Pfizer, Roche, Takeda and TTY Biopharm; and support for attending meetings from AstraZeneca, Roche, and Chugai Pharmaceutical. Other authors declared no conflict of interest.

## ETHICS STATEMENT

This study was approved by the NTUH Research Ethics Committee (201905056RIFD) and all patients provided informed consent prior to enrollment.

## Supporting information


Figure S1.

Figure S2.

Table S1.

Table S2.

Table S3.

Data S1.

## Data Availability

The data that support the findings of this study are available on reasonable request from the authors.
