# Supplementary material for: Tissue or liquid rebiopsy? A prospective study for simultaneous tissue and liquid NGS after first‐line EGFR inhibitor resistance in lung cancer
Source: Cancer Med. 2023 Dec 22;13(1):e6870. doi: 10.1002/cam4.6870 (PMC10807591; doi:10.1002/cam4.6870)
Supplement: Supplementary file 1 — Figure S1. [file CAM4-13-e6870-s002.pptx]

## Slide 1
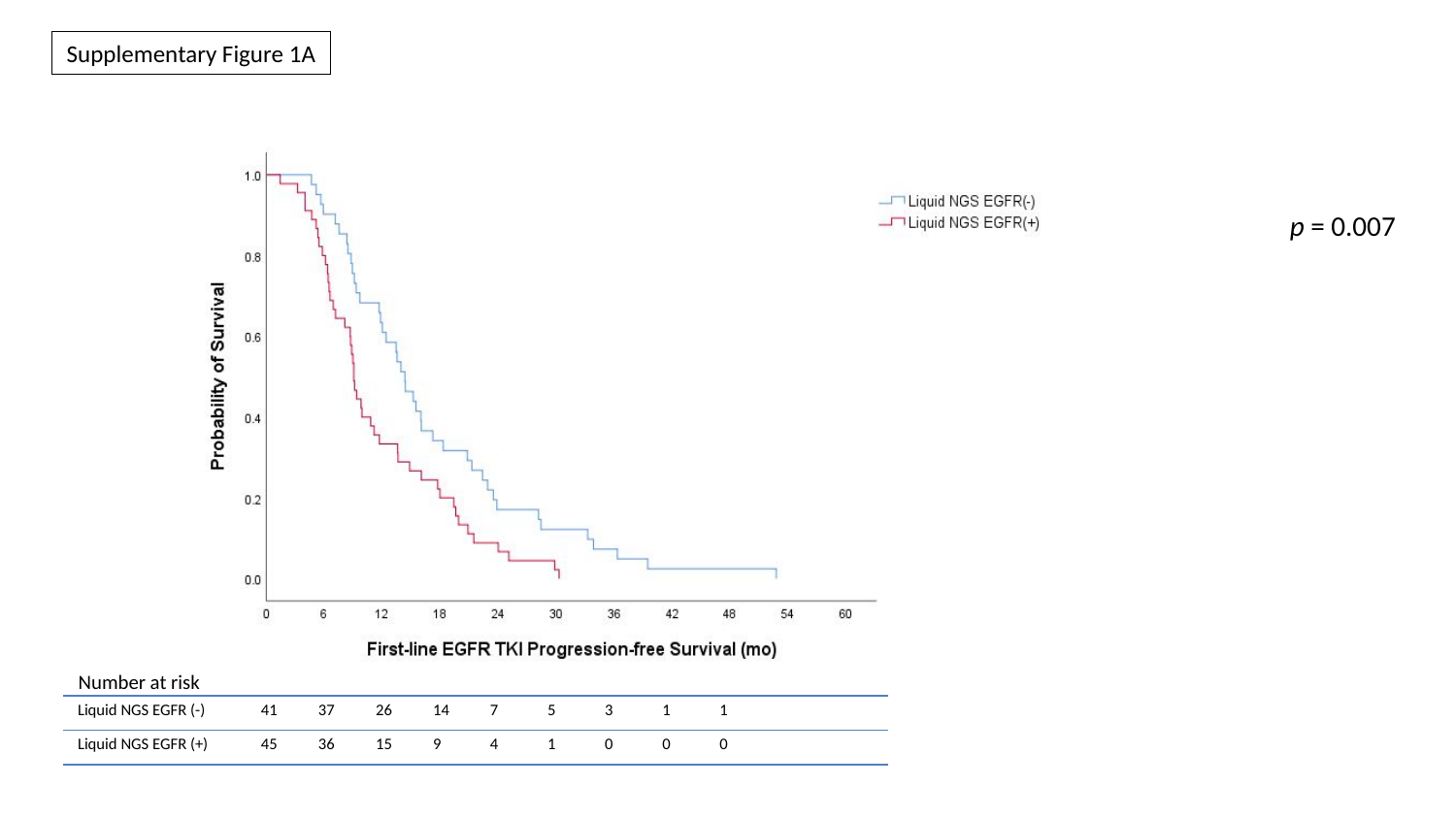

Supplementary Figure 1A
#
p = 0.007
Number at risk
| Liquid NGS EGFR (-) | 41 | 37 | 26 | 14 | 7 | 5 | 3 | 1 | 1 | |
| --- | --- | --- | --- | --- | --- | --- | --- | --- | --- | --- |
| Liquid NGS EGFR (+) | 45 | 36 | 15 | 9 | 4 | 1 | 0 | 0 | 0 | |

## Slide 2
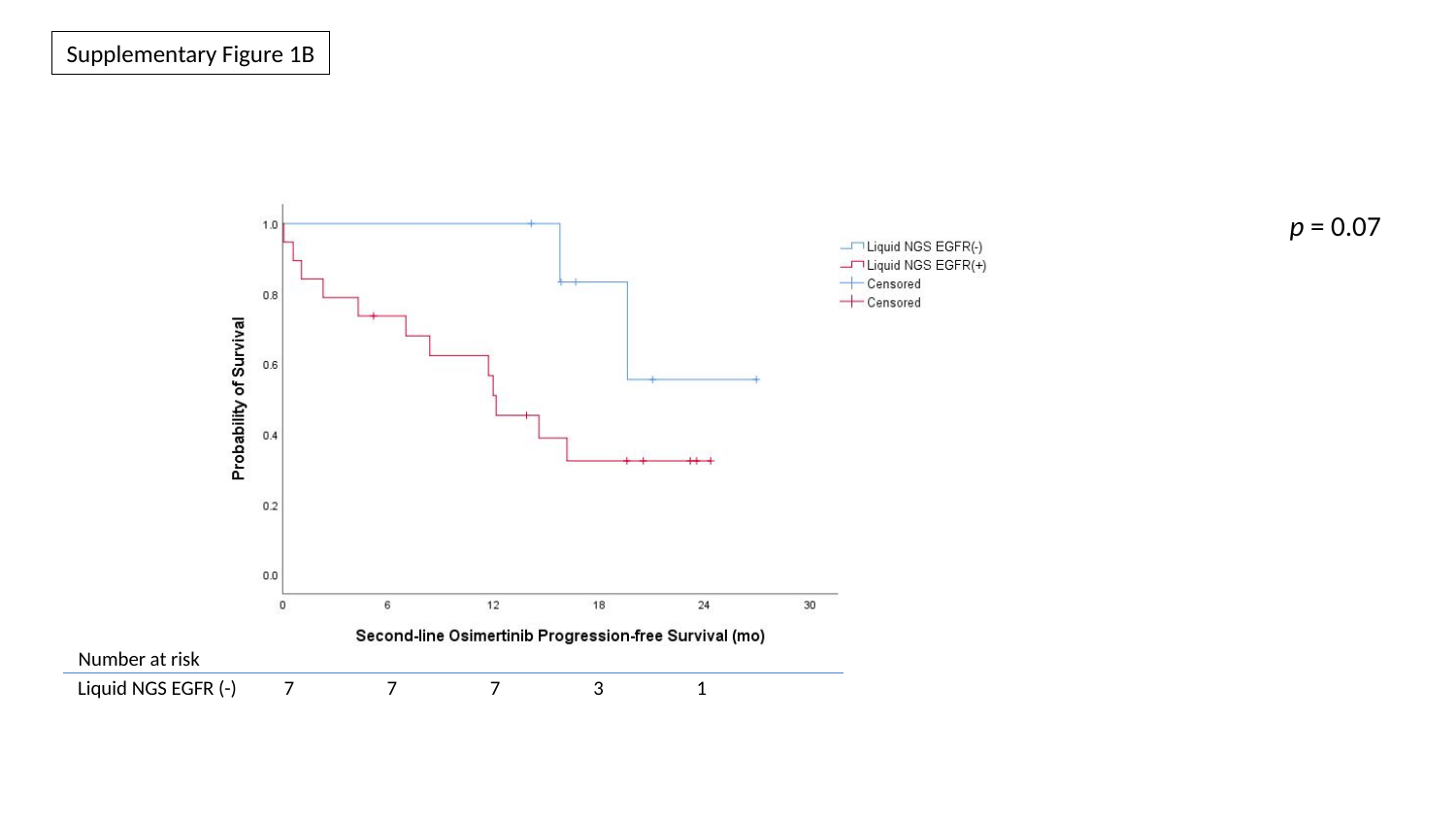

Supplementary Figure 1B
#
p = 0.07
Number at risk
| Liquid NGS EGFR (-) | 7 | 7 | 7 | 3 | 1 | | |
| --- | --- | --- | --- | --- | --- | --- | --- |
| Liquid NGS EGFR (+) | 19 | 13 | 9 | 5 | 1 | | |

## Slide 3
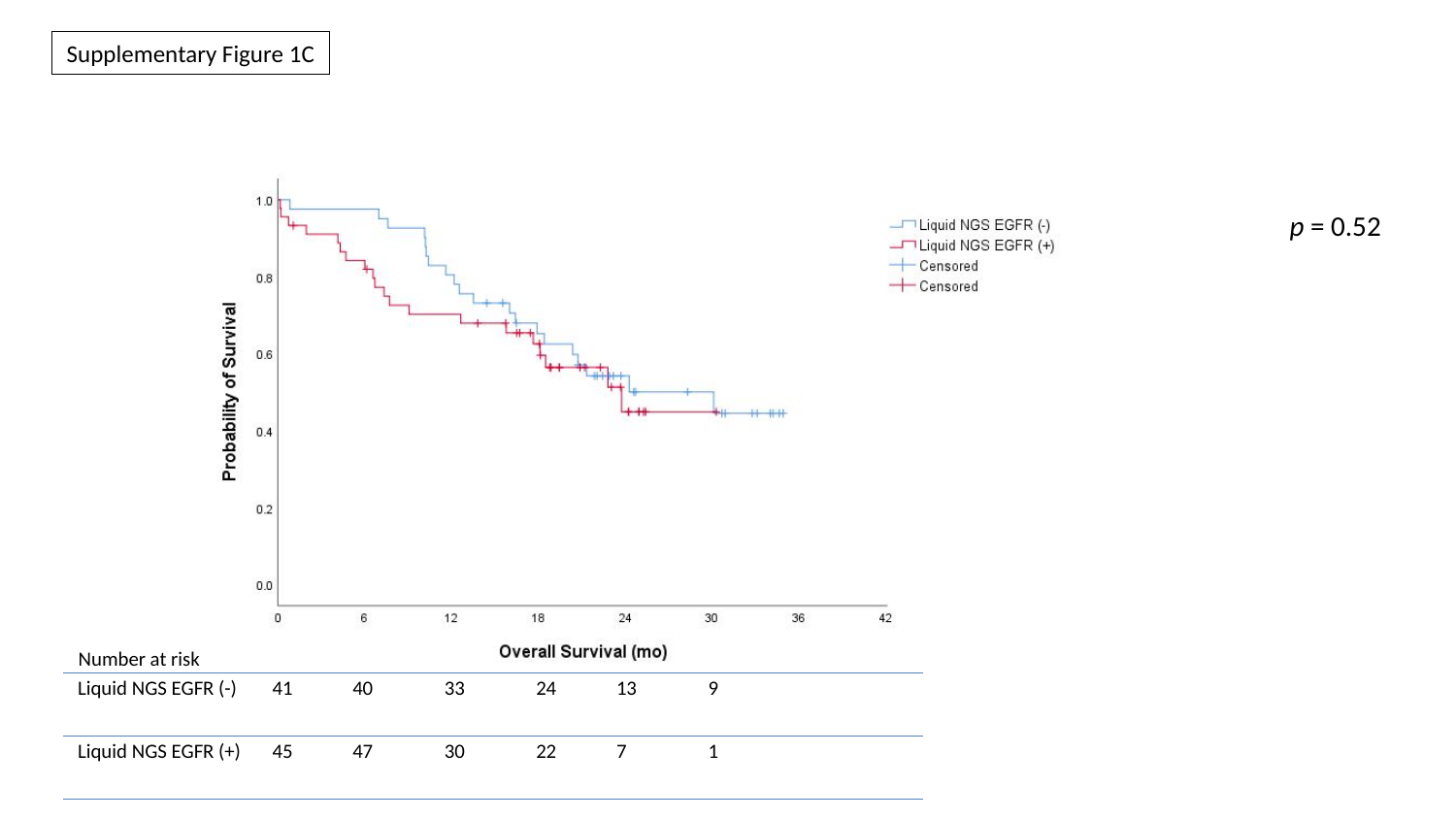

Supplementary Figure 1C
#
p = 0.52
Number at risk
| Liquid NGS EGFR (-) | 41 | 40 | 33 | 24 | 13 | 9 | |
| --- | --- | --- | --- | --- | --- | --- | --- |
| Liquid NGS EGFR (+) | 45 | 47 | 30 | 22 | 7 | 1 | |
