# Supplementary figures and images for: Tissue or liquid rebiopsy? A prospective study for simultaneous tissue and liquid NGS after first‐line EGFR inhibitor resistance in lung cancer
Source: Cancer Med. 2023 Dec 22;13(1):e6870. doi: 10.1002/cam4.6870 (PMC10807591; doi:10.1002/cam4.6870)

## Slide 1
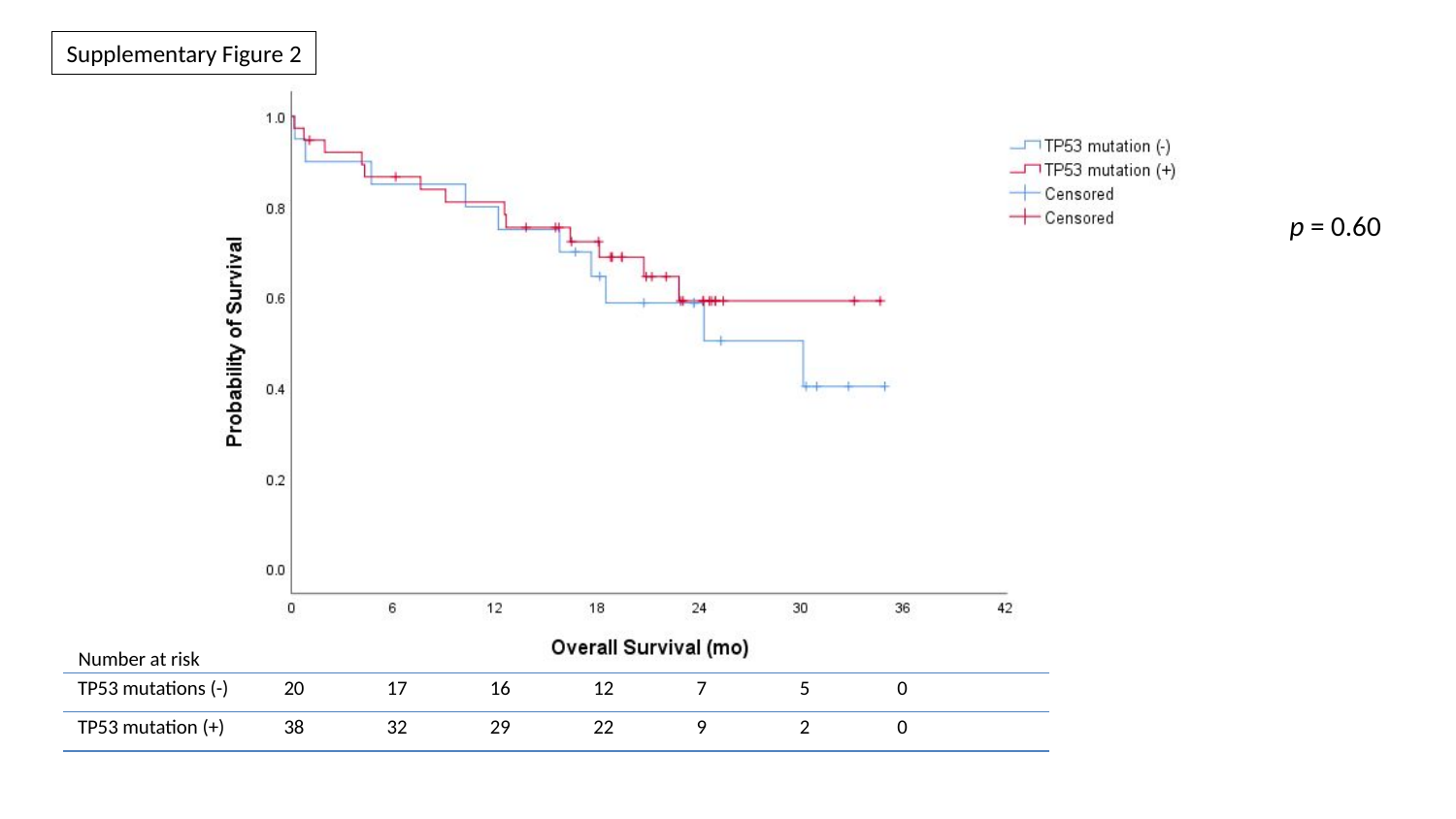

Supplementary Figure 2
#
p = 0.60
Number at risk
| TP53 mutations (-) | 20 | 17 | 16 | 12 | 7 | 5 0 | |
| --- | --- | --- | --- | --- | --- | --- | --- |
| TP53 mutation (+) | 38 | 32 | 29 | 22 | 9 | 2 0 | |

Supplement: Supplementary file 2 — Figure S2. [file CAM4-13-e6870-s006.pptx]
