# Supplementary material for: Tissue or liquid rebiopsy? A prospective study for simultaneous tissue and liquid NGS after first‐line EGFR inhibitor resistance in lung cancer
Source: Cancer Med. 2023 Dec 22;13(1):e6870. doi: 10.1002/cam4.6870 (PMC10807591; doi:10.1002/cam4.6870)
Supplement: Supplementary file 3 — Table S1. [file CAM4-13-e6870-s003.docx]

**Supplementary Table 1. Targeted Gene Lists**


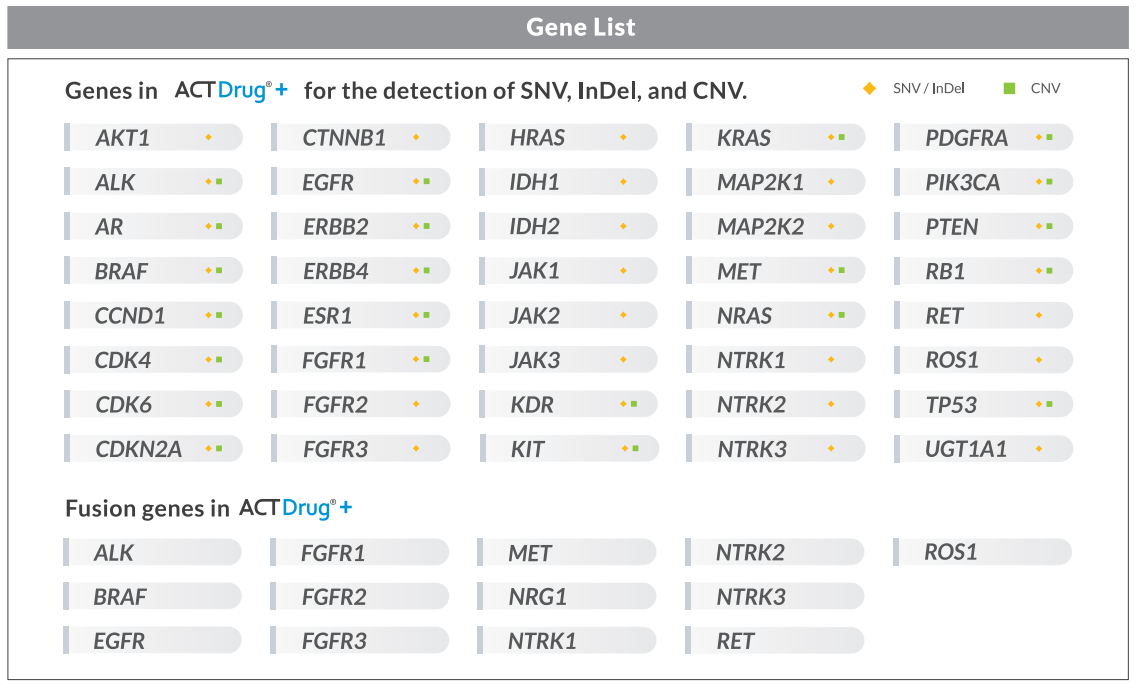


Genes in ACTMonitor^®^Lung for the Detection of SNV and InDels.

| ALK | BRAF | CDKN2A | CTNNB1 | EGFR | ERBB2 |
| --- | --- | --- | --- | --- | --- |
| KRAS | MET | PIK3CA | TP53 | U2AF1 |  |
