# Supplementary material for: Tissue or liquid rebiopsy? A prospective study for simultaneous tissue and liquid NGS after first‐line EGFR inhibitor resistance in lung cancer
Source: Cancer Med. 2023 Dec 22;13(1):e6870. doi: 10.1002/cam4.6870 (PMC10807591; doi:10.1002/cam4.6870)
Supplement: Supplementary file 4 — Table S2. [file CAM4-13-e6870-s005.docx]

| **Supplementary Table 2. Rebiopsy Details (n=86)** | | | |  |  |  |
| --- | --- | --- | --- | --- | --- | --- |
| Variable | Total patients  (n=86) | | Patients with paired NGS results (n=60) | | Patients with  Liquid NGS results only (n=26) | *p* |
|  |  |  |  |  |  |  |
| **Time from progression to tissue rebiopsy**  **(day) (IQR)** | 31 (15-71) | | 32 (14-69) | | 26(16-85) | 0.68 |
| **Rebiopsy attempt for tissue NGS** | 93 | | 63 | | 30 |  |
|  |  | |  | |  |  |
| **Rebiopsy method** |  | |  | |  | 0.02 |
| *Sonography-guided biopsy* | 32 (30%) | | 19 (30%) | | 11 (37%) |  |
| *CT-guided biopsy* | 26 (26%) | | 11 (18%) | | 13 (43%) |  |
| *Bronchoscopy* | 14 (15%) | | 11 (18%) | | 3 (10%) |  |
| *Surgery* | 22 (24%) | | 19 (30%) | | 3 (10%) |  |
| *Pleuroscopy* | 3 (3%) | | 3 (4%) | | 0 (0%) |  |
| **Rebiopsy site** |  | |  | |  | 0.43 |
| *Lung* | 46 (49%) | | 28 (44%) | | 18 (60%) |  |
| *Lymph node* | 10 (11%) | | 6 (10%) | | 4 (13%) |  |
| *Pleura* | 12 (13%) | | 10 (16%) | | 2 (7%) |  |
| *Liver* | 10 (11%) | | 6 (10%) | | 4 (13%) |  |
| *Brain* | 1 (1%) | | 1 (1%) | | 0 (0%) |  |
| *Bone* | 4 (4%) | | 4 (6%) | | 0 (0%) |  |
| *Others* | 10 (11%) | | 8 (13%) | | 2 (7%) |  |
|  |  | |  | |  |  |
|  | |  | |  |  |  |

Abbreviations: NGS, next-generation sequencing; IQR, interquartile range; CT, computed tomography.
