# Supplementary material for: Tissue or liquid rebiopsy? A prospective study for simultaneous tissue and liquid NGS after first‐line EGFR inhibitor resistance in lung cancer
Source: Cancer Med. 2023 Dec 22;13(1):e6870. doi: 10.1002/cam4.6870 (PMC10807591; doi:10.1002/cam4.6870)
Supplement: Supplementary file 5 — Table S3. [file CAM4-13-e6870-s001.docx]

| Variable | EGFR mutation detected by liquid NGS (n=45) | EGFR mutation not detected by liquid NGS (n=41) | *p* |
| --- | --- | --- | --- |
|  |  |  |  |
| **Median age at advanced disease**  **(year-old) (IQR)** | 63.9 (57.4-71.8) | 64.3 (57.8-73.2) | 0.82 |
| **Male** | 16 (36%) | 16 (39%) |  |
| **Patients who never smoked** | 36 (80%) | 35 (85%) | 0.58 |
| **Adenocarcinoma** | 40 (89%) | 37 (90%) |  |
| **Primary EGFR mutation** |  |  | 0.24 |
| *Exon 19 deletion* | 22 (49%) | 15 (37%) |  |
| *L858R* | 21 (47%) | 24 (59%) |  |
| *G791X* | 0 (0%) | 1 (2%) |  |
| *L861Q* | 2 (4%) | 0 (0%) |  |
| *G724S+S768I* | 0 (0%) | 1 (2%) |  |
| **First-line EGFR TKI** |  |  | 0.81 |
| *Gefitinib* | 8 (18%) | 10 (24%) |  |
| *Erlotinib* | 24 (53%) | 19 (47%) |  |
| *Afatinib* | 11 (24%) | 11 (27%) |  |
| *Osimertinib* | 2 (4%) | 1 (2%) |  |
| **Paired tissue and liquid NGS** | 38 (84%) | 22 (54%) | 0.002 |
| **T790M detected** | 26 (58%) | 8 (20%) | <0.001 |
| **2^nd^-line Osimertinib for T790M(+)** | 19 (42%) | 7 (17%) | 0.02 |
|  | | | |

**Supplementary Table 3. Demographic Data and Treatment Outcome between Patients with and without EGFR Mutation Detected by Liquid NGS at Progression (n=86)**

Abbreviations: NGS, next-generation sequencing; IQR, interquartile range; TKI, tyrosine kinase inhibitor.
