# Supplementary material for: Tissue or liquid rebiopsy? A prospective study for simultaneous tissue and liquid NGS after first‐line EGFR inhibitor resistance in lung cancer
Source: Cancer Med. 2023 Dec 22;13(1):e6870. doi: 10.1002/cam4.6870 (PMC10807591; doi:10.1002/cam4.6870)
Supplement: Supplementary file 6 — Data S1. [file CAM4-13-e6870-s004.docx]

**Supplementary method**

**Details of targeted next-generation sequencing (NGS) in this study**

**ACTDrug^®^+** includes a DNA NGS which sequences 40 potential actionable genes and an RNA NGS, ACTFusion^TM^, capable of detecting 13 fusion genes. All patients with adequate rebiopsied cancer tissue in this study (n=60) underwent tissue NGS by **ACTDrug^®^+**.

**ACTDrug**

Tumor purity was checked from the formalin fixed and paraffin embedded (FFPE) cancer tissue. Microdissection was performed, if necessary, to increase tumor purity. After DNA extraction, we amplified the genomic DNA using primer pairs targeting the coding exons of the targeted genes (Supplementary Table 1). Amplicons were ligated with barcoded adaptors and barcoded libraries were subsequently developed. Sequencing was performed using an Ion Proton or Ion S5 sequencer (Thermo Fisher Scientific). Raw reads were mapped to the hg19 reference genome using Ion Torrent Suite (version 5.10). The Variant Effect Predictor (VEP) (version 100) was used to annotate every variant using Clinvar (version 20210208), COSMIC v.92, and Genome Aggregation database r2.1.1. Variants with coverage 25 or more, an allele frequency of 5% or more, and actionable variants with allele frequencies of 2% or more were retained. **ACTDrug** provides uniform coverage of the targeted regions, enabling a target base coverage of 200x more than or equal to 70% with a mean coverage of 800x or more. ONCOCNV (Boeva et al., 2014) was used to normalize the total amplicon number, amplicon GC content, amplicon length, and technology-related biases, followed by segmentation of the sample with a gene-aware model.

**ACTFusion^TM^**

ACTFusion^TM^ is an additional RNA NGS tool capable of detecting 13 fusion genes (Supplementary Table 1) included in the panel of **ACTDrug^®^+**. RNA was extracted from FFPE cancer tissue and transcribed to cDNA using the SuperScript VILO cDNA Synthesis Kit (Invitrogen). The synthesized cDNA was used for library construction. Sequencing was performed using the Ion 540™ Chip/Ion P1™ Chip and Ion GeneStudio™ S5 Prime System/Ion Proton™ System (Life Technologies). **ACTFusion^TM^** provides uniform coverage of the targeted regions, enabling target base coverage at 2000x more than or equal to 70% with a mean coverage of 7000x or more.

**ACTMonitor^®^Lung** was the liquid NGS used in this study. All enrolled patients (n=86) underwent liquid NGS using **ACTMonitor^®^Lung**.

**ACTMonitor^®^Lung**

Cell-free DNA was extracted from patients’ plasma, amplified, and sequenced for the hotpots of the 11 lung cancer-related genes (Supplementary Table 1) using the Ion Torrent sequencing system (Thermo Fisher Scientific). **ACTMonitor^®^Lung** provides uniform coverage of the targeted regions, enabling target base coverage at 2000x more than or equal to 70% with a mean coverage of 7000x or more.
